# Supplementary material for: Dimethyl Fumarate-Loaded Transethosomes: A Formulative Study and Preliminary Ex Vivo and In Vivo Evaluation
Source: Int J Mol Sci. 2022 Aug 6;23(15):8756. doi: 10.3390/ijms23158756 (PMC9369351; doi:10.3390/ijms23158756)
Supplement: Supplementary file 1 [file ijms-23-08756-s001.zip › ijms-1835844-supplementary.pdf]

Dimethyl Fumarate-Loaded Transethosomes: A Formulative Study and Preliminary Ex Vivo and In Vivo Evaluations

Francesca Ferrara[1†], Mascia Benedusi[1†], Franco Cervellati[1], Maddalena Sguizzato[2], Leda Montesi[3], Agnese Bondi[2], Markus Drechsler[4], Walter Pula[2], Giuseppe Valacchi*[1,5,6], and Elisabetta Esposito*[2]

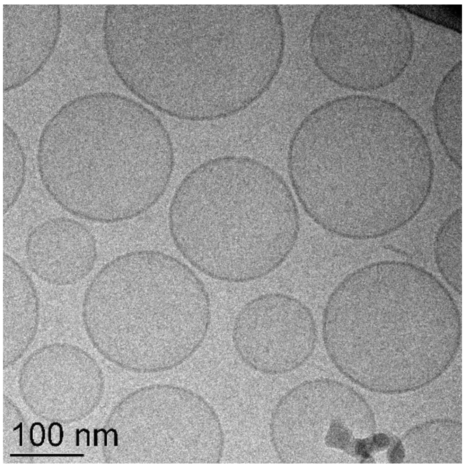

Figure S1. Cryo-TEM image of $TET_{0.9}$-DMF50.

Table S1. Dimensional distribution parameters of $TET_{0.9}$-DMF50, as determined by using PCS and entrapment capacity (EC).

Formulation	Z-Average (nm)	Typical Intensity distribution (nm)	Dispersity index	EC
$TET_{0.9}$-DMF50	140.80 ± 10.50	138.43 (100 %)	0.12 ± 0.04	100.0 ± 1.1

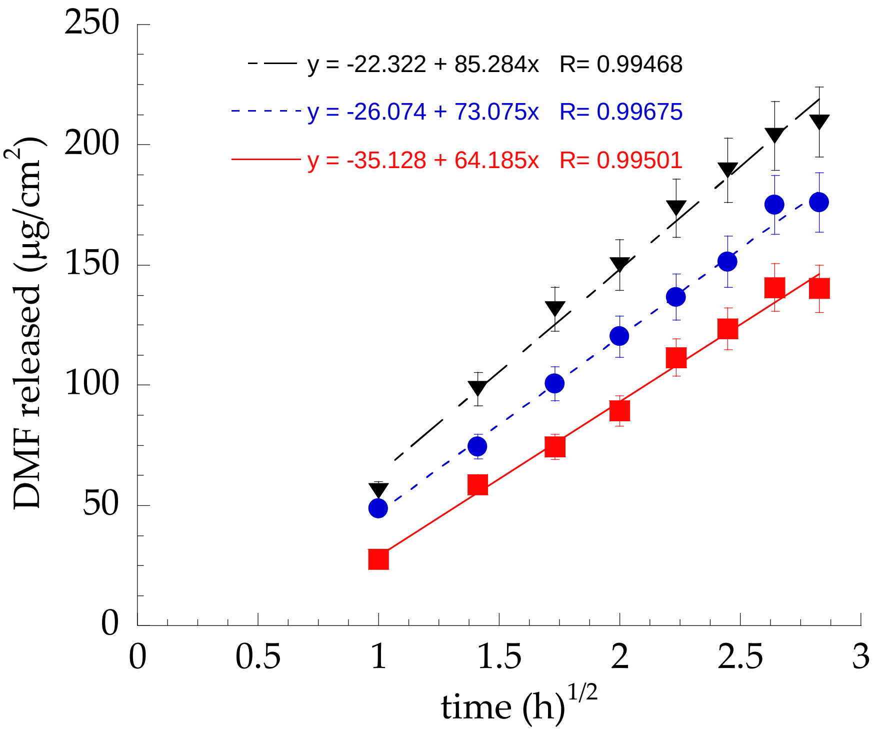

Figure S2. Linear part of DMF release kinetics from $TET_{0.9}$-DMF (●), $TET_{2.7}$-DMF (■), and SOL-DMF (▼), as determined by Franz cell associated with PTFE. Data are the mean of 6 independent experiments ± s.d.

Table S2: IVPT parameters of the indicated forms, as determined by Franz cell associated with Strat-M®.

IVPT parameters	$TET_{0.9}$-DMF50	$TET_{0.9}$-DMF25-gel	$TET_{0.9}$-DMF10-gel
Jss^1 (mg cm^{-2} h^{-1})	1.62 ± 0.02	0.65 ± 0.01	0.17 ± 0.02
T_{lag}^2± s.d. (h)	0.51 ± 0.03	n.p.	n.p.
Kp^3 (cm h^{-1} 10^{-3})	32.48 ± 1.4	26.00 ± 0.51	17.00 ± 0.22
D^4 (cm h^{-1}) x 10^{-3}	0.28 ± 0.10	n.d.	n.d.
$P^5_{membrane/vehicle}$	104.40 ± 5.41	n.d.	n.d.
A_{DMF}^6 (μg cm^{-2})	18.25 ± 12.5	7.50 ± 1.22	2.20 ± 0.88
M_{DMF}^7 (μg cm^{-2})	3.20 ± 0.81	1.22 ± 0.11	0.42 ± 0.13

1: Steady-state flux per unit area, 2: lag time; 3: permeability coefficient; 4: diffusion coefficient; 5: partition coefficient; 6: cumulative amount of DMF diffused at 24 h; 7: DMF associated with the membrane after 24 h; n.p.: not present; n.a. not determined; data are the mean of 6 independent Franz cell experiments ± s.d. Differences statistically significant, $p < 0.05$.
